# Tissue Expression of NGF in Skin Lesions of HIV-Coinfected and Non-Coinfected Leprosy Patients and Its Relationship with Leprosy Neural Damage

**DOI:** 10.3390/microorganisms13102271

**Published:** 2025-09-27

**Authors:** Marília Brasil Xavier, Lucas dos Santos Fontes, Mariana Garcia Borges do Nascimento, Simone Rodrigues dos Passos, Débora Pinheiro Xavier, Larissa dos Santos Alcantara, Elza Baía de Brito, Cláudia Maria de Castro Gomes, Carlos Eduardo Pereira Corbett

**Affiliations:** 1 Research Laboratory in Tropical Dermatology and Endemic Diseases, Nucleus of Tropical Medicine, Federal University of Pará, Belém 66055-240, Brazil; luccasfontes@gmail.com (L.d.S.F.); marianaglborges@gmail.com (M.G.B.d.N.); simonerodpassos@gmail.com (S.R.d.P.); deborapinheiroxavier@gmail.com (D.P.X.); elzabrito999@gmail.com (E.B.d.B.); 2 Infectious Diseases Pathology Laboratory (LIM50), Pathology Department, Medical School, São Paulo University, São Paulo 01246-903, Brazil; larissa.salcantara@gmail.com (L.d.S.A.); gomescla@usp.br (C.M.d.C.G.); ccorbett@usp.br (C.E.P.C.)

**Keywords:** leprosy, HIV, acquired immunodeficiency syndrome, co-infection, neuritis, nerve growth factor, immunohistochemistry

## Abstract

Leprosy remains a significant public health issue, particularly due to its neuropathic consequences, which affect sensory, motor, and autonomic functions, leading to severe disabilities. HIV/AIDS, another major public health concern, overlaps geographically with leprosy and is also associated with peripheral neuropathies, complicating the management of co-infected patients. Understanding how Nerve Growth Factor (NGF) is regulated in leprosy and HIV-leprosy co-infection may contribute to immunomodulatory treatments and neuroimmune response control. A cross-sectional study evaluated NGF tissue expression using immunohistochemistry in 47 HIV/leprosy co-infected patients and 61 leprosy-only patients. The co-infected group had a higher incidence of neuritis (40.4%) and a prevalence of exclusively reversal reactions. However, the occurrence of neuritis was not associated with higher expression of NGF in the tissue. Leprosy reactions were more prevalent in non-co-infected patients with multibacillary forms (50%). Multibacillary forms in both groups of patients showed higher cellular expression of NGF, with a greater tendency for higher NGF expression in non-co-infected multibacillary patients (*p* = 0.0021), suggesting impairment in the immune response involved in the tissue expression of neurotrophins in the co-infected group. Overall, co-infection with HIV did not influence the increase in NGF in the lesions of leprosy patients compared with patients with leprosy alone.

## 1. Introduction

Hansen’s disease, also known as leprosy, is a disease caused by the *Mycobacterium leprae complex* (*M. leprae* and *M. lepromatosis*), both obligate intracellular parasites, incapable of being cultured in artificial media, which have a slow multiplication rate, with a tropism for cells of the skin and peripheral nervous system, and cause the same clinical disease [1,2,3,4]. Transmissibility occurs through the release of the bacillus through the respiratory tract of individuals with a high bacillary load; however, leprosy is not a highly contagious disease [4]. The infected host, through the immune system, has the ability to respond in different ways to the presence of the bacillus, which gives the disease a variety of clinical and histological manifestations [1,2,3], in addition to genetic predispositions that can make the host more susceptible to the appearance of reactive episodes that aggravate the disease [5,6]. Even with great advances in studies and developments in the management of the disease and effective treatment, leprosy continues to be a serious global public health problem, with high detection rates in countries such as India, Brazil, and Indonesia, being classified as a neglected tropical disease [3,4].

Neuropathy caused by the action of the bacillus has long-term consequences, especially related to the loss of nerve function in the face, hands, and feet, and early diagnosis and treatment are necessary to avoid irreversible neural damage. The involvement encompasses the three modalities of the peripheral nervous system: sensory, motor, and autonomic functions [7], and can generate serious sequelae, ranging from loss of protective sensitivity to motor changes. Sensory/motor involvement can lead to ulcerations, bone resorption, and muscle paralysis [8,9].

Acquired immunodeficiency syndrome (AIDS), caused by the human immunodeficiency virus (HIV), is also a disease that has a major impact on public health due to the significant number of new cases. In 2023, 1.3 million people became newly infected with HIV, and in total, by the end of 2023, there were around 39.9 million people living with HIV in the world [10,11]. HIV infection can also lead to peripheral sensory neuropathies, which are the most common neurological complications of this infection, affecting between 10 and 15% of patients with HIV and up to 44% of patients with AIDS, which poses challenges in the management of these conditions. However, other forms of neuropathies are also possible in these patients, such as Inflammatory Demyelinating Polyradicoloneuropathy, Dideoxynucleoside Toxic Neuropathy, Mononeuropathy Multiplex and Autonomic Neuropathy [12,13,14].

Understanding the co-infection of these two diseases is relevant in areas where the two diseases overlap, since it has already been mentioned that leprosy can behave as a disease of immune restoration associated with the immunological improvement of patients upon initiation of highly active antiretroviral treatment (HAART), which impacts the prevalence of leprosy disease in patients living with HIV, increasing the prevalence of paucibacillary forms and even type I reaction episodes [15,16,17]. Furthermore, its symptomatic manifestation may occur as part of the Immune Reconstitution Inflammatory Syndrome (IRIS), which is an inappropriate inflammatory response to an infection, usually occurring in the first 6 months of HAART introduction [18,19,20,21], leading to neurological changes that may add to the changes already common to HIV infection, such as sensory changes, decreased strength, and peripheral neuropathies [22,23].

Previous studies have shown that patients co-infected with leprosy and HIV are more likely to develop early neural damage at the onset of the disease when associated with the period of immunological restoration when compared with patients with isolated leprosy [21] and co-infection between these diseases may represent a factor of worse prognosis of muscle function when compared with patients with one or the other disease in isolation [24].

One way to monitor nerve functions during the course of diseases is through biomarkers that signal the production of the myelin sheath by Schwann cells, such as nerve growth factor (NGF), a neurotrophin first described by Rita Levi-Montalcini, in 1953 [25]. This neurotrophin acts in the maintenance and preservation of sympathetic and sensory neurons and also participates in modulating the sensitivity of peripheral fibers to pain and heat [25,26,27]. Studies indicate NGF [28,29,30], as well as the presence of its direct autoantibody (anti-NGF) [31], as possible indicators of neural damage in leprosy according to its expression. Understanding the regulation of NGF levels in different conditions of the disease and working on anti-NGF levels may be relevant for immunomodulatory treatment and for controlling neuroimmune reactions in leprosy [32,33,34,35].

This study aimed to quantify the expression of NGF in skin lesions of patients with leprosy, with and without HIV co-infection, and to establish relationships with the different variables that characterize the nerve damage caused by the disease.

## 2. Materials and Methods

### 2.1. Study Design

This is a comparative cross-sectional study of two groups of patients diagnosed with leprosy, one without HIV and the other co-infected with HIV, and followed at the dermatology outpatient clinic of the Nucleus of Tropical Medicine of the Federal University of Pará (NMT/UFPA), located in the city of Belém, capital of the state of Pará, Brazil.

### 2.2. Inclusion Criteria

Patients aged 18 to 70 years diagnosed with leprosy were included. To constitute group 1, patients diagnosed with leprosy according to signs and symptoms recommended by the Ministry of Health [4] and complemented by additional tests (slit-skin-smear bacilloscopy and histopathology).

To constitute group 2 of this research, cases of co-infection were considered, with individuals previously diagnosed as HIV positive through serological screening (ELISA) and confirmatory (Western Blot) tests, according to the Brazilian Ministry of Health [36], whether or not they were treated with HAART.

### 2.3. Study Procedures

#### 2.3.1. Data Collection

Sociodemographic and clinical data were collected to characterize the sample. Gender, age, occurrence of neuritis, occurrence of leprosy reaction, type of leprosy reaction presented, and number of nerve trunks affected by palpation were identified according to the Simplified Neurological Assessment, following the standards of the Brazilian Ministry of Health [4]. The Simplified Neurological Assessment is an instrument for assessing neural damage and classifying the Degree of Physical Disability, in which clinical complaints, signs and symptoms, nerve palpation, strength assessment, and sensory inspection and assessment are evaluated.

The clinical classification of leprosy followed the classification criteria of Ridley and Jopling (1966) [1,2], later grouped according to the WHO Operational Classification [7], including the leprosy clinical forms Primary Neural (NP), Indeterminate (I), Tuberculoid (TT), and Borderline Tuberculoid (BT), classified as Paucibacillary Forms (PB); and the forms Borderline Borderline (BB), Borderline Lepromatous (BL), and Lepromatous (LL), classified as Multibacillary Forms (MB) [1,2].

From the group with leprosy/HIV, specific clinical and laboratory data on the follow-up of HIV disease were also collected, including use of HAART, the temporal relationship between the use of HAART and the diagnosis of leprosy, the quantification of CD4+ T lymphocytes per cubic millimeter of peripheral blood at the time of leprosy diagnosis, and if patients had more or less than 350 CD4+ T lymphocytes per cubic millimeter of peripheral blood [36].

#### 2.3.2. Collection of Material for Immunohistochemistry

A skin biopsy was performed, collected from the leprosy lesion at the time of diagnosis, using a number 4 punch after antisepsis and local anesthesia with 2% lidocaine. The material obtained was stored in transparent glass vials with 10% buffered formalin, embedded in paraffin, and sent to the Laboratory of Pathology of Infectious Diseases (LIM50) of the Medical School of the University of São Paulo (FMUSP).

#### 2.3.3. Immunohistochemistry Technique

The stored material was subjected to 4 µm sections and fixed on slides. For immunostaining of the NGF neurotrophin, the Polymer Conjugated Method with Secondary Antibodies immunohistochemical method was used, following the protocol of Hsu et al. (1981) [37], with a methodology partially modified according to Quaresma et al. (2006) [38].

Histological sections were deparaffinized in xylene for 20 min and subsequently hydrated in alcohol baths in decreasing concentrations (twice in absolute alcohol and once in 70% alcohol) for 3 min each. Endogenous peroxidase was blocked with 10 washes of 3 min each with 10% hydrogen peroxide. Next, antigen retrieval was performed in a steam cooker at 96 °C for 30 min, using citrate buffer pH6. After antigen retrieval, the slides were cooled to room temperature until a temperature of 55 °C was reached.

Immediately after, nonspecific sites were blocked. To do this, the slides were incubated in an oven at 37 °C with a 6% skim milk solution in PBS for 30 min. Then, the primary antibody (Anti-NGF antibody ab6199—Abcam, Cambridge, MA, USA) was diluted in 1% bovine albumin in PBS (1:250), pipetted onto the slides, and incubated in a humid chamber for 18 h.

Subsequently, the slides were washed with PBS Tween 0.05% 3 times for 5 min and then incubated in a humid chamber at 37 °C with the post-primary antibody (NOVOLINK TM polymer detection systems—RE7280-K Leica Biosystems, Newcastle, UK) for 30 min. After the incubation time, the slides were washed again with PBS Tween 0.05% 3 times for 5 min and then incubated with the polymer from the same NOVOLINK kit mentioned above for 30 min. After this step, the slides were washed again with PBS Tween 0.05% 3 times for 5 min and then incubated with the chromogenic substrate DAB+H2O2 (diaminobenzidine with hydrogen peroxide— K3468 —AgilentDako, Santa Clara, CA, USA) for five minutes for antigen-antibody binding visualization. Then, the slides were washed in distilled water and counterstained with Harris hematoxylin.

Finally, the slides were dehydrated in baths with increasing concentrations of alcohol (once in 70% alcohol and twice in absolute alcohol) for 2 min each and mounted with coverslips and synthetic resin.

#### 2.3.4. Quantitative Analysis of Immunostained Cells

The immunostained sections were analyzed, and quantification of immunostained cells was performed using AxioVision 4.8.2 software (Zeiss, San Diego, CA, USA) with a 40x A-plan objective lens. The AXIO IMAGER Z1-ZEISS system (Zeiss, Oberkochen, Germany), consisting of a binocular microscope and an automatic photography system with an attached Axiocam camera, was used to document and obtain photomicrographs. Five to eight different fields were randomly selected in the area of the dermal inflammatory infiltrate of the histological lesions. Once the number of stained cells was determined, the average number of cells in the different fields was obtained, with the results expressed in cells per field. The density of positive cells was then calculated, which is the result of the average number of immunostained cells per field divided by the area of the photomicrograph (0.0356412 mm^2^), with the result expressed in the density of stained cells per square millimeter.

### 2.4. Statistical Analysis

The results obtained during the experiments were stored in electronic spreadsheets using Microsoft Office Excel 2007 and analyzed using Biostat 5.0 and GraphPad Prism 10.2. Numerical variables were analyzed by obtaining measures of central tendency such as mean and median, as well as measures of variability such as standard deviation and variance. To verify the statistical significance of the frequency presented by the groups, according to the variable under analysis, the chi-square test of adherence and the g-test were used. To compare the means of NGF expressions between the groups and clinical variables, the *t*-test was used. To verify the correlation between the expressed values of NGF and TCD4+ lymphocytes, the Spearman correlation coefficient was used. The images were created using GraphPad Prism 10.2 and InkScape 1.2.

### 2.5. Ethical Aspects

This study was developed in accordance with the precepts of the standards for research involving human beings, established by Resolution 466/12 of the National Health Council and Law 14.874/2024 of Brazilian legislation. All individuals participating in this research were evaluated after being informed about the research and signing the informed consent form.

This research is linked to the research project entitled “Contributions to the Knowledge of the Immunopathology of HIV/Leprosy Co-infection and HIV-Leishmaniasis Co-infection”, approved by the Research Ethics Committee of the Nucleus of Tropical Medicine of the Federal University of Pará with CAAE 10935419.5.0000.5172 and Opinion Number 3.285.553. This study follows the STROBE (STrengthening the Reporting of OBservational studies in Epidemiology) guidelines.

## 3. Results

A total of 108 participants were selected and divided into two groups: (1) Co-infection Group, consisting of 47 patients with a mean age of 40.04 (±11.35) years, predominantly male (n = 33/70.2%) and with paucibacillary forms of the disease (n = 30/63.8%); and (2) Non-Co-infection Group, consisting of 61 patients with a mean age of 43.75 (±15.87) years, predominantly male (n = 37/60.7%), and with multibacillary forms of the disease (n = 40/65.6%).

At the time of diagnosis, the co-infected group had a higher incidence of neuritis, both for the paucibacillary and multibacillary forms. In general, type I and II leprosy reactions were more prevalent in patients with multibacillary forms in the group not co-infected with HIV. The group of co-infected patients, in turn, presented exclusively type I reactions. Upon nerve palpation, it was observed that, regardless of the clinical form or co-infection, most patients had up to 3 nerve trunks affected (Table 1).

**Figure 1:**
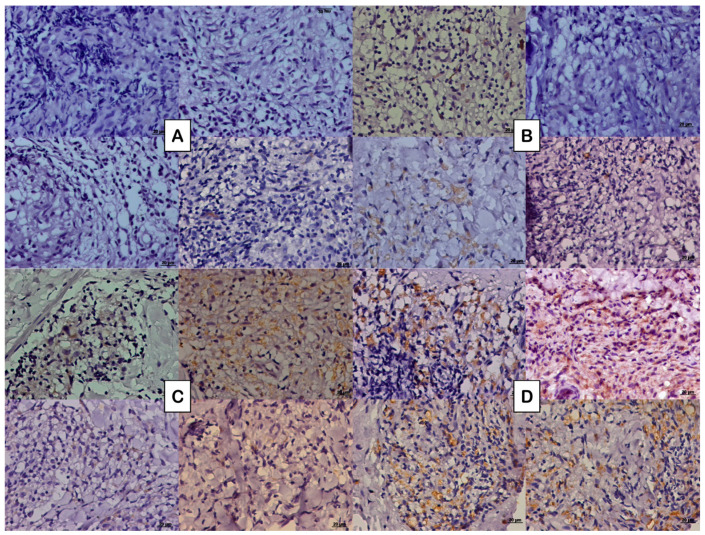
Photomicrograph of the tissue expression of NGF represented by the DAB brownish coloration observed in the cytoplasm of some cells in (**A**) paucibacillary coinfected patients, (**B**) paucibacillary non-coinfected patients, (**C**) multibacillary coinfected patients, and (**D**) multibacillary non-coinfected patients.

**Figure 2:**
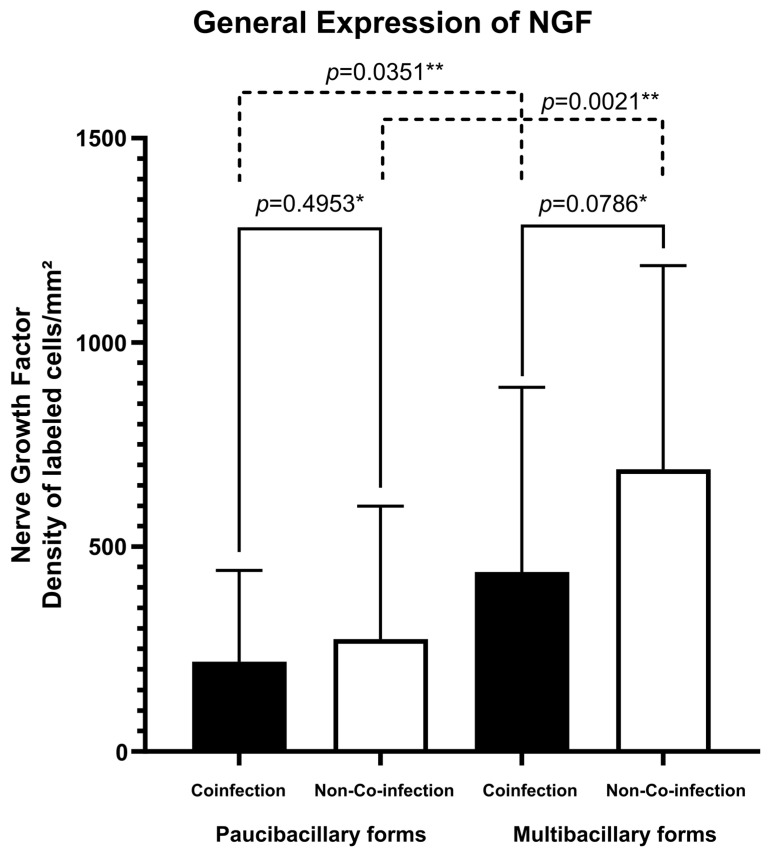
Tissue expression of NGF according to the presence of co-infection and clinical classification. * *p*-value according to the *t*-test in an intragroup analysis. ** *p*-value according to the *t*-test in an intergroup analysis.

Regarding the expression of the NFG neurotrophin, multibacillary forms of both groups of patients presented greater cellular expression (273.77 ± 325.16 labeled cells per square millimeter for co-infected patients and 689.22 ± 498.54 labeled cells per square millimeter for non-co-infected patients) (Figure 1B,D) compared to paucibacillary forms of each group (218.21 ± 222.73 labeled cells per square millimeter for co-infected patients and 437.23 ± 452.30 labeled cells per square millimeter for non-co-infected patients) (Figure 1A,C). The fact of being co-infected with HIV did not change this significant expression (Figure 2). No difference was observed in the spatial distribution of cells immunostained with NGF when comparing co-infected patients with non-co-infected patients.

**Figure 3:**
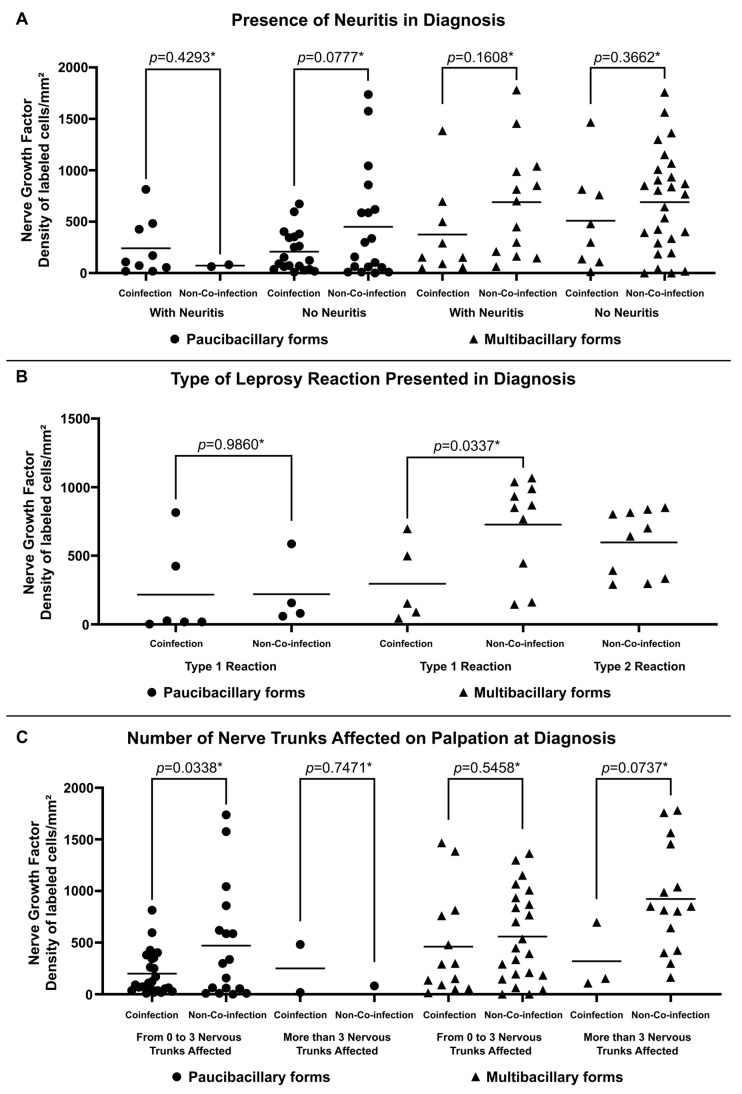
Tissue expression of NGF at diagnosis according to (**A**) Presence of Neuritis in Diagnosis, (**B**) Type of Leprosy Reaction in Diagnosis and (**C**) Number of Nerve Trunks Affected on Palpation at Diagnosis. * *p*-value according to the *t*-test in an intragroup analysis.

Higher expression of NGF was observed in non-co-infected multibacillary patients regardless of the occurrence of neuritis (688.38 ± 534.82 labeled cells per square millimeter for patients with neuritis and 689.62 ± 490.71 labeled cells per square millimeter for patients without neuritis) (Figure 3A). Similarly, multibacillary patients not co-infected with HIV present higher expression of NGF considering the presence of reactions (726.20 ± 348.45 labeled cells per square millimeter for Type 1 Reactions and 596.40 ± 239.97 labeled cells per square millimeter for Type 2 Reactions) (Figure 3B) and the number of altered nerve trunks on palpation (558.05 ± 439.07 labeled cells per square millimeter for patients with 0 to 3 nervous trunks affected and 921.90 ± 517.29 labeled cells per square millimeter for patients with more than 3 nervous trunks affected) (Figure 3C).

In an analysis of the leprosy and HIV co-infection group, it was found that, regardless of the clinical form, the majority were using HAART at the time of diagnosis and had a CD4+ T lymphocyte count of less than 350 cells per cubic millimeter. When the time of use of antiretroviral therapy and the diagnosis of leprosy were correlated, it was found that most leprosy diagnoses occurred within the first six months of starting antiretroviral therapy (Table 2).

**Figure 4:**
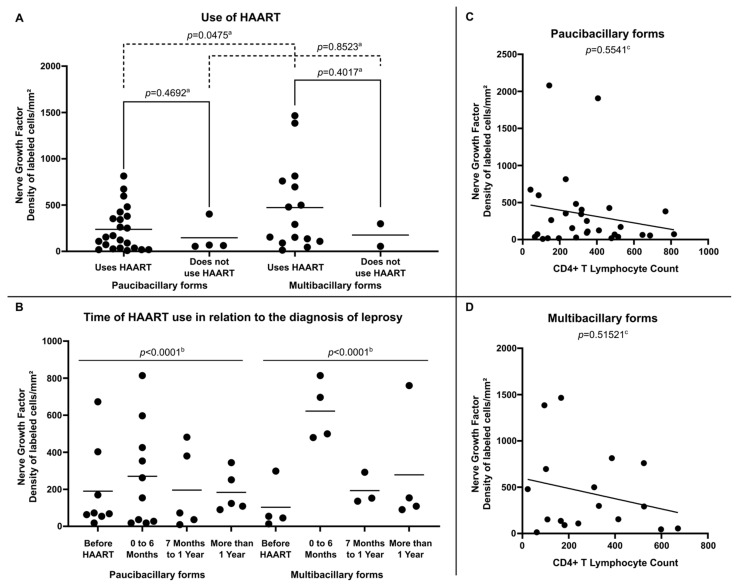
(**A**) Tissue expression of NGF at diagnosis according to Use of HAART, (**B**) Tissue expression of NGF at diagnosis according to Time of HAART use in relation to the diagnosis of leprosy, (**C**) Correlation between NGF tissue expression and CD4+ T Lymphocyte count per cubic millimeter of peripheral blood in Paucibacilary Forms and (**D**) Correlation between NGF expression and CD4+ T Lymphocyte count per cubic millimeter of peripheral blood in Multibacilary Forms. ^a^ *p*-value achieved according to the *t*-test. ^b^ *p*-value according to the Chi-square test of adherence. ^c^ *p*-value achieved according to the Spearman Correlation Coefficient.

Internal analysis in the group of HIV-positive leprosy patients demonstrated that NGF expression was more relevant in multibacillary clinical forms, especially among those using HAART (472.91 ± 469.69 labeled cells per square millimeter) (Figure 4A) and who were diagnosed with leprosy in the period suggestive of immune restoration (in the first six months after starting therapy) (622.30 ± 161.06 labeled cells per square millimeter) (Figure 4B). Regarding the analysis of NGF expression related to the number of CD4+ T lymphocytes in peripheral blood, no significant correlation was observed between these variables in either paucibacillary (Figure 4C) or multibacillary patients (Figure 4D).

## 4. Discussion

Co-infection of leprosy with HIV is a challenge in several aspects [39]. Xavier [15], in 2006, described a series of cases in Brazil in which 31 cases were followed and a prevalence of paucibacillary forms and the occurrence of neuritis were observed during the period of immunological restoration associated with the initiation of HAART [40]. Mouchard and collaborators [21] described, in 2022, a series of cases of co-infected patients, pointing out similar findings. Furthermore, the same authors conducted a systematic review of the literature in which they analyzed 73 patients, predominantly men, with paucibacillary clinical forms and who presented a prevalence of type I leprosy reactions. The observation of two comparative cohorts of leprosy patients coinfected with HIV and another without co-infection, demonstrated similar results and incidence of type 1 leprosy reaction with neuritis [16]. These results reaffirmed data previously suggested in the literature that HIV did not change the polarization of the disease to its malignant pole and presents itself as a disease associated with immunological restoration after the start of HAART [41,42,43,44]. The clinical characteristics of the two groups analyzed here at the time of leprosy diagnosis resemble characteristics already reported in the literature.

The main objective of this study was to analyze the expression of NGF in leprosy lesions and observe possible differences between patients coinfected with HIV, thus assuming that the presence of HIV could increase neural damage, affecting the expression of NGF. Nerve growth factor is a widely studied neurotrophin with defined roles in the development, differentiation, maturation, and preservation of nervous tissue, in addition to its role in inflammatory processes, in which it is increased and can act in a pro-inflammatory and anti-inflammatory way depending on the target tissue [27]. In a study comparing tissue levels of NGF in the skin and peripheral nervous tissues of patients with borderline-virchowian leprosy and healthy individuals, Anand et al. [28], in 1994, found lower levels of this neurotrophin in leprosy patients. The authors suggest decreased levels of NGF as a relevant factor in nervous involvement, such as the loss of sensitivity characteristic of leprosy. Another study [45] also detected lower expression of NGF in leprosy patients compared to healthy individuals.

Regarding the antibody to NGF, there are few studies. Anti-NGF antibodies were found in the serum of patients with all forms of leprosy, with a significant decrease after the use of oral Cyclosporine for the treatment of type II reaction, suggesting the possibility of the contribution of neurotrophin in the pathophysiology of peripheral neuritis observed in leprosy disease [31]. The authors theorized that the high level of the antibody in leprosy patients could explain the decrease in NGF. High levels of anti-NGF antibodies have been detected in inflammatory diseases that produce nerve damage, such as lupus, thyroiditis, and rheumatoid arthritis. However, the relationship between NGF expression and anti-NGF antibodies still appears unclear, and, therefore, studies are still needed to clarify these relationships in diseases of immunological origin [27].

HIV/leprosy co-infection has been shown to be a factor that increases sensory and motor neural damage. Novais et al. [24] analyzed motor alterations in three groups of patients—(1) patients with HIV, (2) patients co-infected with HIV and leprosy, and (3) patients with leprosy without any other comorbidity—and observed a reduction in bilateral strength levels in the co-infected group in relation to the groups of patients with isolated diseases. The sum of sensory and motor damage could be justified by alterations in neurotrophin.

Anand [46] suggested that the decrease in NGF levels in leprosy patients would be an early change in the course of the disease and could be related to the loss of pain sensitivity even with intense tissue inflammatory activity, which could be related to the modulatory activity of neurotrophin on sensitivity in peripheral fibers to pain and heat [26].

The results of this study do not allow comparing leprosy patients co-infected with HIV and non-co-infected with healthy controls. However, by establishing similarities and the occurrence of greater neural damage at the time of diagnosis in leprosy patients also carrying HIV, due to the inflammatory state and sum of nerve damage [13,20], it was expected to find greater expression of NGF in co-infected patients than in patients not co-infected with HIV. However, the results presented here suggest that being a carrier of HIV did not significantly change the expression of NGF. This fact can be justified by the higher prevalence of multibacillary forms in non-co-infected patients and of paucibacillary forms in co-infected patients, according to the paradox of leprosy in patients living with HIV [15,39], and, since, as reported in previous studies, multibacillary forms present higher expression of this neurotrophin [35,47]. The multibacillary clinical form was the factor associated with the greater expression of this neurotrophin in both groups. The results found in this study, indicating a higher expression of NGF in lesions of leprosy patients with multibacillary forms, corroborate studies that compared the expression of NGF among leprosy patients with different clinical forms of the disease, with a higher expression of neurotrophin in patients with multibacillary forms [35,47], indicating an attempt to promote greater neuroprotection by increasing neurotrophin expression and consequent tissue remodeling and regeneration [27,48,49]. This pattern was also observed in other cytokines, such as TGF-β [35,50]. This could indicate a relationship between NGF expression and a Th2 inflammatory response pattern, which involves the expression of cytokines such as IL-4 and IL-10, greater nervous involvement, and greater inflammatory activity, characteristic of multibacillary forms of the disease [51].

The analysis of possible differences between neural damage in patients co-infected and non-co-infected with HIV was carried out by Xavier et al. [22] in 2018. A higher incidence of neuritis was observed in patients co-infected at the time of diagnosis, associated with the period of immunological restoration, with a slightly higher probability of developing sensory damage, especially in patients with multibacillary forms of the disease. However, the group of co-infected leprosy patients showed a significantly faster improvement in symptoms during treatment with MDT when compared to the group of leprosy patients without HIV. This would be justified by the fact that the non-co-infected group had a higher prevalence of multibacillary forms, with reactions with more prolonged neuritis, indicating that the neural damage in the co-infected group, despite being early and suggesting some influence from possible damage associated with HIV, tends to be reversible and follow, after immune restoration, the natural course of neural involvement caused by the leprosy bacillus [22].

Several studies have evaluated the role of neurotrophins, especially NGF, and suggest that they have a notable role in nerve damage, with expression in both neural and non-neural tissues, and have confirmed their function and that of their receptors as a marker of nerve injury, an adjuvant in regeneration, and in the activation/sensitization function of nociception [52]. It is postulated that the increase in expression in multibacillary forms in relation to other forms is due to the intensity of inflammatory reactions, immunological reactions towards the Th2 pole, and high bacillary load with a greater number of lesions and affected nerves, culminating in more prominent sensory alterations. The results found here showed a greater tendency for greater expression of NGF in non-co-infected multibacillary forms, considering the occurrence of neuritis, occurrence of reactions, and a greater number of affected nerve trunks, even when compared to co-infected multibacillary forms. Considering that patients with HIV, even during the period of immunological restoration, present intense immunological modifications with alterations in CD4+ lymphocytes, quantitatively and qualitatively, what would justify the difference in the inflammatory response and the difference in neurotrophin expression, and could it be one of the reasons for the non-elevation of NGF as expected [53,54,55].

Analyzing the expression of NGF only in the group of leprosy patients with HIV, it was observed that this was more relevant in multibacillary clinical forms, especially among those who used HAART and who were diagnosed with leprosy in the period suggestive of immune restoration (in the first six months after starting therapy), which corroborates the assumption that immune restoration favors neurotrophin production [53,56].

Levels of peripheral CD4+ lymphocytes did not correlate with the expression of NGF in the tissue. It is known that the tissue response is compartmentalized, and other studies have already demonstrated that levels of peripheral CD4+ lymphocytes are not related to the profile of CD4+ lymphocytes and tissue cytokines [15,57].

The main limitation of the study is the number of patients analyzed, since leprosy has a wide range of clinical manifestations within the pauci-multibacillary spectrum. However, in the context of leprosy-HIV co-infection, there is still a shortage of patients reported in the literature, and these are restricted to areas of significant geographic overlap, such as the northern region of Brazil. Considering that co-infection of leprosy with HIV is still prevalent in areas of high endemicity for both diseases, investigations should be conducted to better understand the immunopathology of nerve damage caused by this co-infection.

The importance of this study is due to its contribution to the immunopathological knowledge of leprosy and leprosy-HIV co-infection. Therefore, in regions with geographic overlap of these diseases, where there may be a sum of neural damage caused by each of them [22], it is important that possible biological markers, such as NGF, be studied so that they can be used as predictors of early neural damage, thus enabling the establishment of protective measures and measures to minimize this damage in patients. Further studies should be carried out with this marker so that this discussion can evolve.

The clinical and immunopathological characteristics of peripheral nerve damage in patients with concomitant HIV infection still have many gaps. It is necessary to consider that paucibacillary forms differ clinically and immunologically, and analyses should be made from this perspective. In addition, based on this study, other immunostainings should be performed to better understand the immunopathology of leprosy and leprosy-HIV co-infection.

## Figures and Tables

**Table 1:** Clinical characteristics of patients in the study population.

Variables	Number of Observations (%)				*p*-Value
	Paucibacillary (N = 51)		Multibacillary (N = 57)		
	Co-Infection (n = 30)	Non-Co-Infection (n = 21)	Co-Infection (n = 17)	Non-Co-Infection (n = 40)	
**Neuritis in diagnosis**					
Presence	10 (33.3)	2 (9.5)	9 (52.9)	13 (32.5)	*p* < 0.0001 *
Absence	20 (66.7)	19 (90.5)	8 (47.1)	27 (67.5)	*p* = 0.0039 **
**Leprosy reactions in diagnosis**					
Presence	6 (20)	4 (19)	6 (35.3)	20 (50)	*p* = 1.0000 *
Absence	24 (80)	17 (81)	11 (64.7)	20 (50)	*p* = 0.0364 **
**Type of Leprosy Reaction**					
Type I	6 (100)	4 (100)	6 (100)	10 (50)	*p* = 1.0000 *
Type II	0	0	0	10 (50)	*p* < 0.0001 **
**Number of nerve trunks affected on palpation ^a^**					
From 0 to 3 Trunks	26 (92.9)	18 (96.7)	13 (81.2)	23 (60.5)	*p* = 0.5271 *
More than 3 Trunks	2 (7.1)	1 (5.3)	3 (18.8)	15 (39.5)	*p* = 0.0009 **

* g-test performed intragroup between Paucibacillary Coinfected and Non-Coinfected patients, ** g-test performed intragroup between Multibacillary Coinfected and Non-Coinfected patients. ^a^ For the variable “Number of nerve trunks affected on palpation”, the information available from 47 patients in the “Paucibacillary Group” and 54 patients in the “Multibacillary Group” were used.

**Table 2:** Clinical characteristics related to HIV in patients in the Co-infection Group.

Variables	Number of Observations (%)		*p*-Value
	Co-Infection (N = 47)		
	PB (n = 30)	MB (n = 17)	
**Use of HAART**			
Using HAART	24 (80)	15 (88.2)	*p* = 0.0819 *
Not using HAART	6 (20)	2 (11.8)	
**Time of HAART use in relation to the diagnosis of leprosy**			
Before HAART	9 (30)	4 (23.6)	*p* = 0.1373 *
0 to 6 months	11 (36.6)	5 (29.4)	
Between 6 and 12 Months	5 (16.7)	3 (17.6)	
More than 1 Year	5 (16.7)	5 (29.4)	
**CD4+ T Lymphocyte Dosage on peripheral blood**			
<350 cells/mm^3^	19 (63.4)	11 (64.7)	*p* = 1.0000 *
>350 cells/mm^3^	11 (36.6)	6 (35.3)	

* *p*-value according to the g-test performed intragroup between Paucibacillary and Multibacillary Coinfected Patients.

## Data Availability

The original contributions presented in this study are included in the article. Further inquiries can be directed to the corresponding author.

## Abbreviations

The following abbreviations are used in this manuscript:

AIDS	Acquired Immunodeficiency Syndrome
HIV	Human Immunodeficiency Virus
HAART	Highly Active Antiretroviral Treatment
NGF	Nerve Growth Factor
NP	Primary Neural
I	Indeterminate
TT	Tuberculoid
BT	Borderline Tuberculoid
PB	Paucibacillary Forms
BB	Borderline Borderline
BL	Borderline Lepromatous
LL	Lepromatous
MB	Multibacillary Forms
STROBE	STrengthening the Reporting of OBservational studies in Epidemiology
